# The Blockade of IL6 Counterparts the Osmolar Stress-Induced Apoptosis in Human Conjunctival Epithelial Cells

**DOI:** 10.1155/2016/8350134

**Published:** 2016-07-31

**Authors:** Hee-Jung Ju, Yong-Soo Byun, Jee-Won Mok, Choun-Ki Joo

**Affiliations:** ^1^Catholic Institute for Visual Science, Seoul St. Mary's Hospital, Seoul 06591, Republic of Korea; ^2^Department of Ophthalmology, Seoul St. Mary's Hospital, College of Medicine, The Catholic University of Korea, Seoul 06591, Republic of Korea

## Abstract

To determine the effect of hyperosmolarity on cell survival/apoptosis of conjunctival epithelial cells and evaluate the possible role of IL6, Wong-Kilbourne derivative of Chang conjunctival cell line (WKD) was used in this study. Confluent cells were incubated under different osmolarity (290 mOsm and 500 mOsm) with or without neutralizing IL6 antibody (50 ng/mL). The expression of IL6 level was measured in the supernatant of each conditioned medium. Cell viability/apoptosis assay was performed using Annexin V/Propidium Iodide (PI) and Cell Counting Kit-8 (CCK-8). Western blot was conducted to measure the abundance of apoptotic markers and IL6 related downstream signaling pathway. The concentration of IL6 showed time-dependent increase in cells treated with 500 mOsm. Although apoptosis of WKD cell is increased in treated 500 mOsm for 24 h, apoptosis reduced in WKD cell treated 500 mOsm with anti-IL6 for 24 h. Anti-IL6 inhibited the activation of JAK-STAT signaling pathway, which was induced by hyperosmolarity. Hyperosmolar condition induced apoptosis in conjunctival epithelial cells, along with increase of IL6 production. IL6 neutralizing antibody inhibited apoptosis and JAK-STAT signaling in hyperosmolar condition. These findings suggested that IL6 may be involved in apoptotic change and in hyperosmolarity.

## 1. Introduction

Dry eye syndrome is a multifactorial disease, which is caused by a vicious cycle: abnormalities of tear film and lacrimal hyposecretion induce the break-up of tear film, and the following inflammation of ocular surface deteriorates the secretion and the composition of tears [1]. Hyperosmolarity is induced by lacrimal hyposecretion or the increase of evaporation evokes desquamation, decreased intercellular connections, blunting and loss of microplicae, cell membrane disruption, and cellular swelling with decreased cytoplasmic density in the corneal epithelium [2]. Moreover, hyperosmolarity provokes squamous metaplasia, loss of goblet cells, and inflammation in the conjunctival epithelium [3–5]. These phenomena decrease the production of mucin for lubricating corneal epithelium, and the reduction of mucin aggravates dry eye [6]. Histologic findings of dry eye in patients with Sjögren syndrome and immunosuppressant patients, such as postbone marrow transplantation state, were reported as a reduction of goblet cells, increment of inflammatory cells in cornea and conjunctiva, and inflammation with fibrosis of the lacrimal gland [7–10]. Dry eye also induces the secretion of cytokines such as IL6, IL-1*β*, TNF-*α*, and IFN-*γ* [11]. Cyclosporin, one of the drugs suppressing those cytokines, reduces the infiltration of conjunctival cells and IL6, regulates the necrosis of conjunctival epithelial cells, and elevates the number of goblet cells by preventing the loss of the cells [12].

IL6 has been known as a representative cytokine with increased expression in tears and the conjunctival epithelium of eyes with dry eye syndrome. It has also been reported to have pro- and anti-inflammatory effects. As evidence of the proinflammatory effects, a report revealed that IL6 treatment reduced the survival of liver cancer cells [13] and tocilizumab, an IL6 blocker, has been used as a therapeutic drug for autoimmune diseases in rheumatology. Anti-inflammatory effects of IL6 were revealed by the following evidence: IL6 treatment on cells increased migration [14], IL6 induced cell migration and wound healing of mouse biliary epithelial cells [15], blocking of IL6 reduced inflammatory-related molecules in mouse alkali burn model [16], and IL6-deficient mice showed delayed wound healing after skin resection [17].

Although IL6 has been well known as an important cytokine on disease progression and severity associated with immune mechanisms, its role in dry eye was still vague except for increased expression on the ocular surface. Therefore, this research investigated the role of IL6 on apoptosis of conjunctival epithelial cells after hyperosmolarity.

## 2. Material and Methods

### 2.1. In Vitro Osmolar Stress Experiment

The Wong-Kilbourne derivative of Chang conjunctival cells (WKD, ATCC CCL-20, Manassas, VA, USA) were cultured in Dulbecco's modified Eagle's Medium F12 (1 : 3) culture medium (Invitrogen, Waltham, MA, USA), supplemented with 1% penicillin and streptomycin (WELGENE, Daegu, Seoul) and 5% heat-inactivated fetal bovine serum (WISENT, Quebec, Canada). When cells were approximately 80–90% confluence, culture medium was replaced with fresh medium with added 1 M NaCl to increase the osmolarity (corresponding to 290, 500 mOsm) for 24 h. Cells were incubated for 24 h before protein extraction and conditioned medium collection.

The blockade of IL6 was done 24 h before incubation using the neutralizing Anti-Human IL6 antibody (anti-IL6, Clone 1936, R&D systems, Minneapolis, MN, USA) for neutralizing the IL6. To determine whether IL6 cytokine has protected effect in the NaCl exposure or not, conjunctiva epithelial cells were incubated with IL6 for 24 h and then exposed to 500 mOsm NaCl for 24 h.

### 2.2. Measurement of Cytokine Production

 Cell supernatant was collected 290 mOsm, 500 mOsm NaCl incubation after 24 h, and then centrifuged 12,000 rpm for 3 min at 4°C. Cytokine levels in supernatant were determined using the Human IL-1*β*, IL6, TNF-*α*, and IFN-*γ* Quantikine Enzyme-Linked Immunosorbent Assay Kit (R&D systems, Minneapolis, MN, USA) and we followed the manufacturer's protocol. Briefly, for IL6 kit 100 *μ*L of standard and samples were incubated in antibody-coated plate at 2 hrs. After being washed four times, add 200 *μ*L conjugate solution being incubated at 2 hrs. After being washed four times, add 200 *μ*L substrate solution being incubated at 20 min. Add 50 *μ*L stop solution and read at 450 nm with reader within 30 min.

### 2.3. Cell Viability Assay

Cell viability was determined using the Cell Counting Kit-8 (Dojindo, Kumamoto, Japan) according to the manufacturer's instructions. Cells (1 × 10^5^ cells/mL, 100 *μ*L) were seeded in 96-well plate. When cells were confluent 80%–90%, cells were treated with anti-IL6 for 24 h. After treatment for 24 h, add 10 *μ*L of CCK-8 solution and incubate the plate for 1 h and cover the plate to protect from light, and then measure the absorbance using a microplate reader at 450 nm. The experiment was performed three times.

### 2.4. Flow Cytometry

Cells were seeded at 1.5 × 10^6^ cells in a well of 60 mm plates and cultured until confluent 80–90%. Cells were exposed to 290 mOsm, 500 mOsm NaCl with/without anti-IL6 50 ng for 24 h. After 24 h, cells were collected and resuspended. For labeling the Annexin V/Propidium Iodide (PI), we were using the Annexin V/PI kit (Invitrogen, Eugene, Oregon, USA). The suspension cells were labeled with Annexin V/PI and analyzed by flow cytometry (BD, Franklin, New Jersey).

### 2.5. Western Blot Assay

Cells were harvested by scraping with RIPA buffer. Extracts were incubated for 2 h at 4°C and obtained by centrifugation (13,000 rpm for 20 min at 4°C). Protein concentrations were determined using the BCA assay Kit (Thermostat, Hercules, CA, USA), and whole-cell extracts were adjusted to same amount of total protein (20 *μ*g). Samples were electrophoresed in 10% SDS-PAGE. Proteins were then transferred onto a PVDF membrane (Millipore Corporation, Billerica, MA, USA) at 300 mA for 90 min at 4°C, and the membranes were incubated with 3% BSA (Sigma-Aldrich, St. Louis, MO, USA) in TBST to block nonspecific binding. Primary antibodies were incubated over night at 4°C. And we washed five times with TBST (0.5% tween 20 in 1x TBS) the secondary antibodies conjugated horseradish peroxidase (HRP) (Santa Cruz, Dallas, Texas, USA) that was applied and incubated for 1 h at RT. After five times of washing with TBST (0.5% tween 20 in 1x TBS), the membrane followed by chemiluminescent detection using Immobilon Western Substrate (Millipore Corporation, Billerica, MA, USA) with the ChemiDoc MP Imaging system (Bio-Rad Laboratories Inc., Hercules, California, USA). The antibodies diluents were shown at Table 1.

### 2.6. Statistical Analysis

All results were indicated as means ± SEM. The results were analyzed by Kruskal-Wallis analysis, followed by a Mann-Whitney analysis. A *p* value of less than 0.05 was considered to be statistically significant.

## 3. Results

### 3.1. Hyperosmolarity Induces IL6 Levels in the WKD Cells

**Figure 1:**
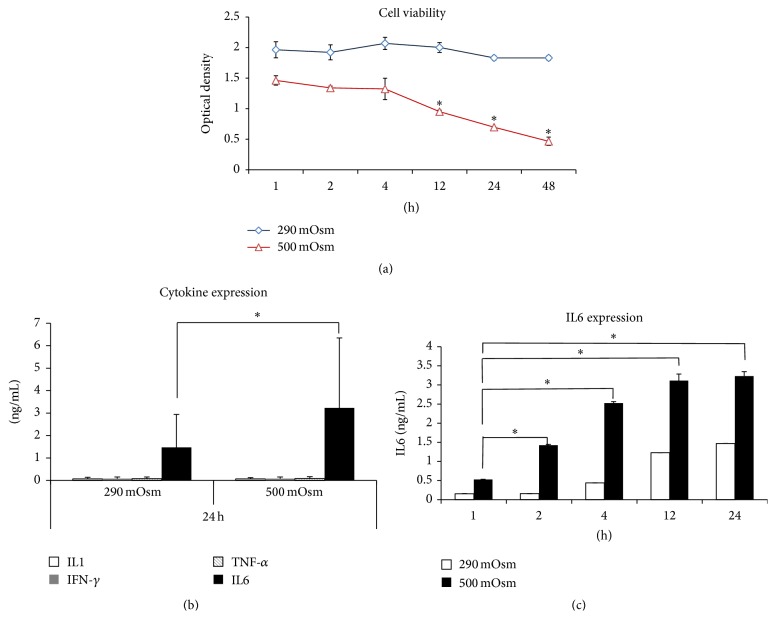
Change of conjunctival epithelial cells under 290 mOsm and 500 mOsm conditions. (a) Cell viability was significantly decreased in 500 mOsm at 12, 24, 48 h compared to 290 mOsm. (b) Cytokine ELISA assay showed 500 mOsm was significantly increased compared to 290 mOsm in cell supernatant. (c) IL6 increased depending on the time course, and the expression was higher in 500 mOsm than that in 290 mOsm. ^*∗*^ *p* = 0.05, *n* = 3.

WKD cells were cultured under 290 mOsm and 500 mOsm for 24 h and the cell viability was analyzed. There were no definite apoptotic cell deaths in 290 mOsm, while cell viability was significantly reduced after 12 h in 500 mOsm (0.95 ± 0.04 at 12 h, 0.69 ± 0.012 at 24 h, and 0.47 ± 0.12 at 48 h, resp., *p* = 0.05 at 12 h, 24 h) (Figure 1(a)). We assessed the levels of IL-1*β*, TNF-*α*, IFN-*γ*, and IL6 from medium of cells cultured under 290 mOsm and 500 mOsm to identify the inflammatory cytokines which are well known to increase in epithelial cells by hyperosmolarity stress. Quantitative analysis showed that, whereas IL-1*β* (0.07 ± 0.002 in 290 mOsm, 0.06 ± 0.002 in 500 mOsm), TNF-*α* (0.08 ± 0.004 in 290 mOsm, 0.08 ± 0.0004 in 500 mOsm), and IFN-*γ* (0.08 ± 0.009 in 290 mOsm, 0.08 ± 0.003 in 500 mOsm) showed no significant change, IL6 (1.47 ± 0.44 in 290 mOsm, 3.23 ± 0.12 in 500 mOsm) were increased (*p* = 0.05, Figure 1(b)). When measuring the time-dependent expression of IL6 in 290 mOsm and 500 mOsm, the expression of IL6 in 500 mOsm increased from 0.012 pg/mL at 1 h to 2.1 pg/mL at 24 h, which was higher than that in 290 mOsm (*p* = 0.05, Figure 1(c)).

### 3.2. IL6 Mediates Hyperosmolarity Induced Apoptotic Cell Death

**Figure 2:**
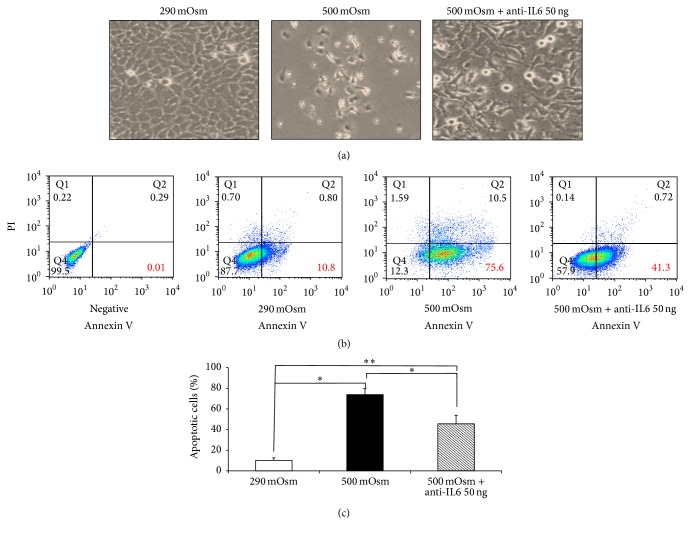
Effect of neutralizing IL6 antibody on cell apoptosis. (a) When evaluating the cell morphology, cell death decreased in 500 mOsm with anti-IL6 treatment. (b) Flow cytometry with Annexin V/PI showed reduced apoptosis under treatment of anti-IL6 and (c) the proportion of apoptotic cells significantly reduced by treatment of anti-IL6. ^*∗*^ *p* = 0.022, ^*∗∗*^ *p* = 0.023, *n* = 3.

To evaluate whether IL6 was secreted to protect cells or induce cell death in apoptosis by hyperosmolarity, 50 ng of anti-IL6 was used for blocking IL6. When investigating change of morphology and cell distributions in 290 mOsm, 500 mOsm, and 500 mOsm with anti-IL6, the cells in 500 mOsm showed shrinkage and more apoptosis than in 290 mOsm. Nevertheless, cells in 500 mOsm with anti-IL6 showed less apoptosis and shrinkage than those in 500 mOsm without anti-IL6 (Figure 2(a)).

We have given a 500 mOsm on the conjunctival epithelial cells and the effect of IL6 was examined using Annexin V/PI. The amount of the cells was significantly increased in the cell pretreated with the anti-IL6. The apoptosis rates are increased from 10.2 ± 2.44 in 290 mOsm to 73.88 ± 5.84 in 500 mOsm and 45.58 ± 2.89 in 500 mOsm NaCl with anti-IL6. Treated anti-IL6 was very effectively decreased for the apoptosis rates in 500 mOsm NaCl treated (Figure 2(b)).

There was significant difference in the proportions of apoptotic cells among 290 mOsm, 500 mOsm, and 500 mOsm with anti-IL6 (Figure 2(c)), which implies that IL6 could promote apoptosis by hyperosmolarity.

### 3.3. Anti-IL6 Inhibits JAK-STAT Pathway Activation in Hyperosmolarity Induced Cell Apoptosis

Evaluating JAK-STAT signaling and apoptosis markers by Western blot, the expressions of p-STAT3, p-ERK1/2, and p-mTOR were higher in 500 mOsm (resp., 1.01 ± 1.16, 1.19 ± 0.22, and 1.15 ± 0.08) than 290 mOsm (resp., 0.43 ± 0.15, 0.44 ± 0.08, and 0.48 ± 0.03); however, those were lower in 500 mOsm with anti-IL6 (resp., 0.6 ± 0.17, 0.72 ± 0.17, and 0.9 ± 0.12) than in 500 mOsm.

**Figure 3:**
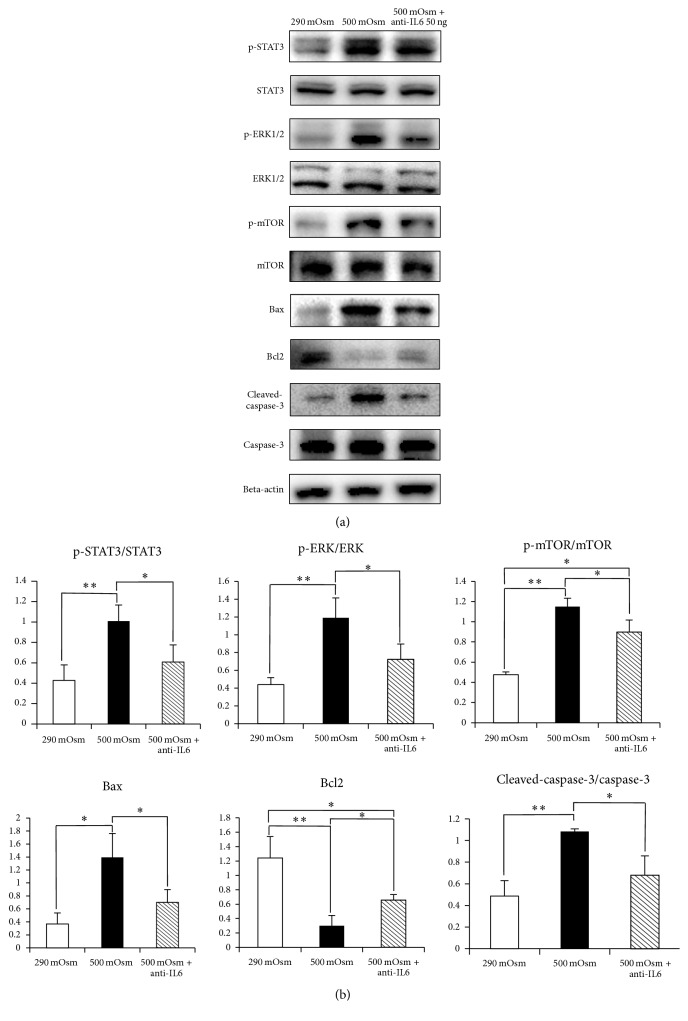
Effect of IL6 on JAK-STAT signaling and apoptosis markers. (a) The factors of the JAK-STAT signaling pathway and apoptosis markers in 500 mOsm and 500 mOsm with anti-IL6 were investigated by Western blot. JAK-STAT signaling was augmented in 500 mOsm and reduced in 500 mOsm with anti-IL6, and apoptosis markers also changed in similar pattern. (b) Those differences between 500 mOsm and 500 mOsm with anti-IL6 were statistically significant in the graph. ^*∗*^ *p* = 0.05, ^*∗∗*^ *p* = 0.05, *n* = 3.

Bax and cleaved-caspase-3 showed higher expression in 500 mOsm (resp., 1.39 ± 0.37, 1.08 ± 0.02) than in 290 mOsm (resp., 0.37 ± 0.17, 0.49 ± 0.14), and those were also lower in 500 mOsm with anti-IL6 (resp., 0.7 ± 0.2, 0.68 ± 0.18) than in 500 mOsm. Bcl2 showed lowest expression in 500 mOsm (0.3 ± 0.14), followed by that in 500 mOsm with anti-IL6 (0.66 ± 0.08) and 290 mOsm (1.24 ± 0.3) (Figure 3(a)). The significant difference of expressions of p-STAT3/total STAT3, p-ERK/total ERK, and p-mTOR/total mTOR was revealed between those in 500 mOsm and 500 mOsm with anti-IL6 (*p* = 0.05) Apoptosis markers such as Bax, Bcl2, and cleaved caspase-3/caspase-3 showed significant discrepancy between those in 500 mOsm and 500 mOsm with anti-IL6 (*p* = 0.05, Figure 3(b)).

## 4. Discussion

It has been reported that hyperosmolarity stress could induce apoptosis and promote the secretion of diverse proinflammatory cytokines [18]. The association between conjunctival epithelial cells and IL6 has been described in many studies: increased expression of IL-1*β*, IL8, IL6, and TNF-*α* in conjunctival epithelial cells and tear fluid in the patients with dry eye syndrome [19, 20] and increase of IL-1*β*, IL8, and, especially, IL6 in impression cytology [21, 22]. Those studies suggested conjunctiva epithelial cells could aggravate dry eye and IL6 had an important role in pathomechanism of dry eye. A previous study showed that blocking IL6 could increase goblet cells in conjunctival epithelium and reduce inflammatory cells, which could soothe the symptoms and prevent the chronic progression of dry eye [12]. Flow cytometry analysis showed that the blocking of IL6 significantly suppressed apoptosis observed at 500 mOsm, which suggests blocking IL6 could modify apoptosis following hyperosmolarity.

Cytokines such as IL6 can activate the JAK-STAT signaling pathway [23]. IL6 can be activated though the membrane-bound IL6 receptor (classical pathway) or the soluble type of IL6 receptor (trans-signaling). The IL6 attached with receptor could initiate cascade via JAK activation, and activated JAK kinase phosphorylate induces forming of dimer by phosphorylation of STAT3 [24, 25]. This signaling pathway promotes proliferation, differentiation, migration, and apoptosis of cells [26]. Although the activation of the JAK-STAT signal by IL6 was observed in cancer [27], there have been few studies for the association between conjunctival epithelial cells and IL6, especially about the role of increased IL6 in a hyperosmolar state of conjunctival epithelial cells. This research revealed that the IL6/JAK/STAT signaling pathway was associated with apoptosis induced by hyperosmolarity. Western blot showed that 500 mOsm promoted the expression of STAT3 (direct signal), ERK, and mTOR (indirect signal), and IL6 reduced the increased expressions. Bax and caspase-3 as apoptosis markers showed higher expression in hyperosmolar state (500 mOsm), which was suppressed by blocking IL6. The inverse pattern of Bcl-2 expression related to IL6 and hyperosmolarity was also revealed. These suggested that JAK-STAT signaling pathway of apoptosis was associated with IL6.

**Figure 4:**
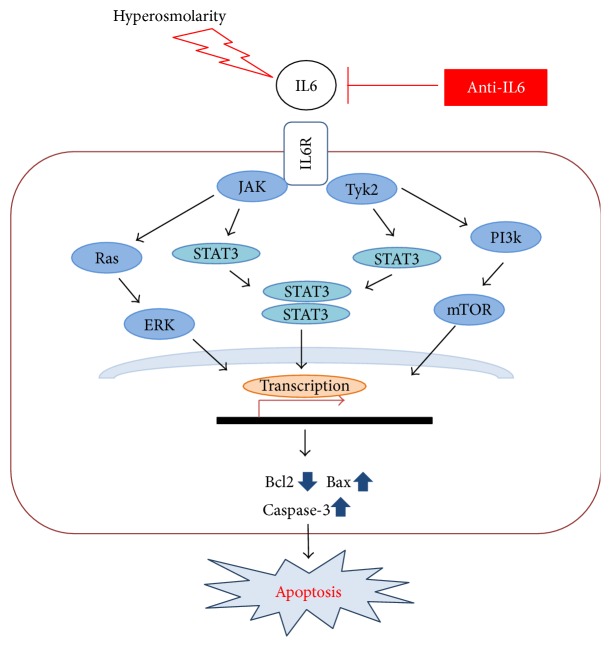
The role of IL6 induced by hyperosmolarity. Hyperosmolarity induced the expression of IL6 followed by apoptosis, whereas blocking IL6 suppressed JAK-STAT signaling and apoptosis.

In this study, blocking IL6 inhibited the apoptosis of conjunctival epithelial cells under hyperosmolar condition, and the process was associated with the JAK-STAT signal pathway (Figure 4). This indicated that IL6 could be one of the important cytokines affecting the pathogenesis of dry eye. Therefore, in dry eye patients, IL6 could be a biologic marker, and regulating IL6 though the IL6/JAK/STAT3 signaling pathway could be an effective therapeutic target.

## 5. Conclusion

Hyperosmolarity induced apoptosis in conjunctival epithelial cells was suppressed by blocking IL6, which suggests that IL6 may play an important role in the pathogenesis of dry eye disease.

## Figures and Tables

**Table 1:** List of antibodies, sources, and dilutions.

	Primary antibody	Host	Dilution	Manufacturer
JAK-STAT signaling markers	p-STAT3	Mouse	1 : 1000	Santa Cruz
	STAT3	Rabbit	1 : 1000	Santa Cruz
	p-ERK1/2	Rabbit	1 : 1000	Santa Cruz
	ERK1/2	Mouse	1 : 1000	Santa Cruz
	p-mTOR	Rabbit	1 : 1000	Santa Cruz
	mTOR	Goat	1 : 1000	Santa Cruz

Apoptosis markers	Bax	Rabbit	1 : 500	Santa Cruz
	Bcl2	Mouse	1 : 500	Santa Cruz
	Cleaved caspase-3	Rabbit	1 : 500	Abcam
	Caspase-3	Mouse	1 : 1000	Santa Cruz

Housekeeping gene marker	Beta-actin	Rabbit	1 : 4000	Abcam
